# Supplementary material for: Quality Assessment of Internet Information Regarding Periodontitis in Persons Living with HIV
Source: Int J Environ Res Public Health. 2024 Jun 29;21(7):857. doi: 10.3390/ijerph21070857 (PMC11276730; doi:10.3390/ijerph21070857)
Supplement: Supplementary file 1 [file ijerph-21-00857-s001.zip › ijerph-3045414-supplementary.pdf]

Supplementary Table S1: Number of hits by search engine according to search terms used.

Combination of Search Terms	Search Engines		
	**Google™**	**Bing™**	**Yahoo®**
**Periodontitis and HIV**	580,000	398,000	2,240,000
**Periodontal disease and HIV**	1,310,000	1,500,000	2,110,000
**Gum disease and HIV**	9,610,000	1,210,000	1,250,000
**Total hits**	20,208,000		
